# 24-Hour Holter Monitoring for Identification of Arrhythmias in Elderly Heart Failure Patients: A Single-Centre Study

**DOI:** 10.7759/cureus.32054

**Published:** 2022-11-30

**Authors:** Mani Teja K, Minakshi Dhar, Khushboo Bisht, Barun Kumar

**Affiliations:** 1 Internal Medicine, All India Institute of Medical Sciences, Rishikesh, Rishikesh, IND; 2 Clinical Pharmacology, All India Institute of Medical Sciences, Rishikesh, Rishikesh, IND; 3 Cardiology, All India Institute of Medical Sciences, Rishikesh, Rishikesh, IND

**Keywords:** 2d echocardiography, arrythmia, ejection fraction, holter, electrocardiography

## Abstract

Introduction

Chronic heart failure (CHF) is a common condition seen in the elderly, and unlike coronary artery disease (CAD), its prevalence increases with age. Electrocardiography (ECG) at rest is a simple, non-invasive investigation that is recommended in the initial evaluation of patients with heart failure. The purpose of this study was to identify various ECG abnormalities in elderly patients with heart failure and to find out whether Holter monitoring increases the chances of identifying arrhythmia in them.

Materials and methods

The current study was a single-centre, cross-sectional observational study. The study was conducted by collecting data from patients with heart failure attending the medicine and cardiology departments of All India Institute of Medical Sciences, Rishikesh, a tertiary care facility, from May 1st, 2020, to October 31st, 2021. The goal of this study was to look for electrocardiographic abnormalities in elderly heart failure patients who had a reduced (<50%) or preserved (>=50%) left ventricular ejection fraction (LVEF). All consecutively admitted patients who fulfilled the inclusion criteria were enrolled in the study after taking informed consent. All patients underwent echocardiography, electrocardiography and Holter monitoring. Demographic parameters were collected on a pre-formed proforma, and data was entered into an Excel sheet.

Results

A total of 101 patients were analyzed, and abnormal ECG results were found in 98% of them. Out of 101 patients, 80 (79.2%) patients had heart failure with reduced ejection fraction (HFrEF), with LVEF < 50%, and 21 (20.8%) patients had heart failure with preserved ejection fraction (HFpEF), with LVEF >=50%. Hypertensive heart disease was the most common etiological factor attributing to heart failure in 37 (36.6%) patients and more among heart failure with reduced EF (p=0.01) followed by ischemic heart disease. Old ischemic changes were the commonest ECG abnormality, found in 48 (47.53%) patients with heart failure and among 45 patients (56.25%) with HFrEF (p=<0.001 ). Atrial fibrillation was the most common arrhythmia, occurring in 25 (24.75%) patients with heart failure. The detection rate of arrhythmia by Holter was better than surface ECG in patients with HErEF (p=0.007).

Conclusion

Electrocardiogram (ECG) in elderly heart failure patients is almost always abnormal. The majority of the patients in our study had more than two ECG abnormalities. ECG is a simple non-invasive tool that can detect underlying etiological factors attributing to heart failure. Holter monitoring can be done as an additional modality to detect arrhythmias in heart patients with reduced ejection fraction for risk stratification.

## Introduction

Heart failure (HF) is a major public health problem which affects 26 million patients worldwide and 3.5 million new patients every year. The burden of heart failure is disproportionately distributed among the elderly. Studies have shown that over half of patients hospitalized with HF are aged more than 75 years. The prevalence of HF is approximately 10% for those above 80 years and less than 1% for those less than 40 years of age [1]. Although the advancement of healthcare delayed the onset of HF and improved the prognosis of the patients, there is an urgent need to integrate geriatric medicine with simple techniques to diagnose and manage heart failure and its complications. Congestive heart failure (CHF) is more common among the elderly, and unlike coronary artery disease (CAD), its prevalence is known to increase with age. Despite a mere 17% of those under the age of 65 being affected, the bulk of interventional research on the treatment for HF has focussed on this small population and extended the findings to the older population. Heart failure symptoms and indicators are comparable in both young and elderly people, however, the elderly are more likely to have a non-specific clinical presentation. For the initial evaluation of patients with HF, simple non-invasive investigation like electrocardiography (ECG) is recommended. This is because the surface ECG and 24-hour Holter monitoring are adequate for detecting many abnormalities that could lead to or worsen heart failure and are easily available in resource-limited settings. In many developing nations, there is a lack of information regarding ECG abnormalities in the elderly with HF. The purpose of this study was to investigate several etiological factors contributing to HF and to identify ECG abnormality/abnormalities in North Indian elderly heart failure patients.

## Materials and methods

The current study was a single-centre cross-sectional observational study that was initiated after approval from the Institutional Ethics Committee, All India Institute of Medical Sciences (AIIMS), Rishikesh, Uttarakhand (AIIMS/IEC/21/483). Study data were collected from patients with a diagnosis of HF who attended the outpatient department of Internal Medicine and Cardiology departments of AIIMS, Rishikesh, a tertiary care facility. The period of study was of 18 months (1.5 years) from 1 May 2020 to 31 October 2021. The objective of this study was to identify etiological factors of HF in the elderly and to investigate electrocardiographic abnormalities in elderly heart failure patients with varying levels of left ventricular ejection fraction (LVEF). A total of 101 patients of age 60 years and above with HF were enrolled. A brief clinical history was obtained and relevant electrographic examinations were performed to confirm the diagnosis of HF. All patients underwent echocardiography and were classified based on left ventricular ejection fraction (LVEF) into three groups such as heart failure with reduced ejection fraction (HFrEF) - ejection fraction (EF) ≤ 40%, heart failure with mid-range ejection fraction (HFmrEF), EF = 41% to 49%, and heart failure with preserved ejection fraction (HFpFE), EF = 50%. All measurements were taken according to the American Society of Echocardiography. Patients who were on antiarrhythmic drugs, antibiotics and antipsychotics which prolong QT prolongation were excluded. Left ventricular hypertrophy (LVH) point score ≥ 5 and left atrial enlargement (LAE) were diagnosed as per Romhilt +Estes criteria and Morris index criteria, respectively. Corrected QT interval (QTc) was obtained by the Bazette formula. Standard definitions of other variables were adopted. All patients underwent 24-hour Holter monitoring by using Medilog FD12 plus device (Schiller, Baar, Switzerland).

IBM SPSS Statistics for Windows, Version 25.0 (IBM Corp., Armonk, USA) was used for statistical analysis. During analysis, HF with EF ≤ 40% and those with EF between 41% and 49% were considered in the HFrEF group. Continuous data are presented as mean ± standard deviation (SD) and categorical data as frequency or percentage. The normality of the data was assessed using the Shapiro-Wilk test/Kolmogorov-Smirnov test. A two-tailed probability value (p-value) of less than 0.05 was considered statistically significant.

## Results

Among 101 patients enrolled in this study, 68 (67.3%) were males and 33 (32.7%) were females. Thus, the elderly male-to-female ratio was 2:1. The mean age of elderly patients was 68.7±7.14 years with ages ranging from a minimum of 60 years to a maximum of 100 years. Hypertension was the main comorbidity in the study population (55 patients, 54.4%) followed by type 2 diabetes mellitus (34 patients, 33.6%) and other comorbidities like hypothyroidism, chronic obstructive pulmonary diseases, obesity, and obstructive sleep apnoea were present (32 patients, 31.7%). 

Table 1 shows the age and sex distribution of study participants. There was no statistically significant age difference between the patients with reduced and preserved ejection fraction (p=0.638), although there were more male patients in the HFrEF group than in the HFpEF, however, the difference was not significant (p = 0.551). 

**Table 1:** Age and sex distribution of study participants LVEF: Left Ventricular Ejection Fraction; n: number of patients

Demographic features		LVEF < 50% (n = 80)	LVEF ≥ 50% (n = 21)	p-value
Age		68.4+/-7.28	67.57+/-6.76	0.638
Sex	Male	55 (68.75%)	13 (61.9%)	0.551
	Female	25 (31.25%)	8 (38.1%)	
Mean LVEF		33%+/-0.08	60%+/-0.02	<0.001

Table 2 shows various etiologies of heart failure. Hypertensive heart disease (HHD - 36.6%) and ischemic heart disease (IHD - 35.6%) were major etiologies among all patients with HF as well as major causes of HF in patients with reduced ejection fraction (p=0.001).

**Table 2:** Etiology of heart failure with LVEF LVEF: left ventricular ejection fraction; n: number of patients; IHD: Ischemic Heart Disease; DCMP: Dilated Cardiomyopathy; HHD: Hypertensive Heart Disease; HOCM: Hypertrophic Cardiomyopathy; RHD: Rheumatic Heart Disease; Others: hypothyroid, anemia, restrictive cardiomyopathy, alcoholic cardiomyopathy

Etiology	LVEF < 50% (n = 80)	LVEF ≥ 50% (n = 21)	TOTAL (n = 101)	p-value, Chi-squared
IHD	32 (40%)	4 (19%)	36 (35.6%)	0.07, 3.183
DCMP	10 (12.5%)	-	10 (9.9%)	-
HHD	23 (28.75%)	14 (66.66%)	37 (36.6%)	0.001, 10.3
HOCM	-	2 (9.5%)	2 (1.9%)	-
RHD	1 (1.25%)	-	1 (0.9%)	-
Others	23 (28.75%)	3 (14.286%)	26 (25.7%)	0.177, 1.82

Table 3 shows various ECG abnormalities in patients with HF. Abnormalities were detected on surface ECG in 99 (98.02%) patients with heart failure. Poor R wave progression and the presence of Q waves were predominant ECG findings in all patients with HF. These findings confirm coronary artery disease as the major cause of heart failure among the North Indian elderly. Arrhythmias were picked up on surface ECGs in 52 cases (51.48%) and 71 cases (70.29%) on 24-hour Holter monitoring as shown in Table 4.

**Table 3:** Electrocardiographic abnormalities in the study population LVEF: left ventricular ejection fraction; n: number of patients; ECG: Electrocardiography; AV: Atrio-ventricular; QTc: Corrected QT; MI: Myocardial infarction

ECG abnormalities	LVEF < 50% (n = 80)	LVEF ≥ 50% (n = 21)	Total (n = 101)	p-value, Chi-squared
Bradycardia or tachycardia	7 (8.75%)	3 (14.28%)	10 (9.9%)	0.44, 0.57
Left axis deviation	25 (31.25%)	4 (19.04%)	29 (28.71%)	0.41,0.67
Left Atrial Enlargement	11 (13.75%)	1 (4.76%)	12 (11.88%)	0.25, 1.28
Left ventricular hypertrophy	12 (15%)	7 (33.33%)	19 (18.81%)	0.05, 3.66
Heart Blocks (AV or Bundle branch)	26 (32.5%)	3 (14.28%%)	29 (28.71%)	0.10, 2.69
QTc Prolongation	15 (18.75%)	5 (23.80%)	20 (19.80%)	0.60, 0.26
ST-T Changes	18 (22.5%)	7 (33.33%)	25 (24.75%)	0.31, 1.04
Atrial Fibrillation	20 (25%)	5 (23.80%)	25 (24.75%)	0.91, 0.01
Old MI Changes (Q waves, poor R-wave progression)	45 (56.25%)	3 (14.28%)	48 (47.53%)	<0.001, 11.75

**Table 4:** Comparison of Surface ECG and Holter in picking up the arrhythmias among the two subgroups ECG: Electrocardiography; LVEF: Left ventricular ejection fraction; n: number of patients

Group		Arrhythmia present	Arrhythmia absent	p-value	Chi^2 ^value
LVEF < 50, n=80	ECG	43	37	0.007	7.87
	Holter	60	20		
LVEF ≥ 50, n=21	ECG	9	12	0.53	0.38
	Holter	11	10		

## Discussion

This observational study was carried out for 18 months during which 101 elderly patients were enrolled. More than two ECG abnormalities were noted in the majority of elderly patients with HF (98.02%), while most of them were males. Thus, North Indian elderly males were found to suffer more from HF compared to females of similar age groups. Holter monitoring showed a higher incidence of arrhythmias when compared to surface ECG in patients with HFrEF (p<0.007). 

We observed that 37 of 101 patients (36.6%) suffered from HHD, which was the commonest etiology of HF in the elderly. This finding was more consistent in the patients who suffered from HFrEF (p=0.001). Followed by HHD, IHD was the second most common etiology of heart failure in both groups (40% in HFrEF and 19% in HFpEF). Studies conducted by Owan et al. [2] and Bhatia et al. [3] found coronary artery disease as the most common cause of HF, whereas studies conducted by Thomas et al. [4] and Opadijo and Omotosho [5] reported HHD as the common cause of HF, which is similar to our study.

Chronic changes of myocardial infarction such as poor progression of R-wave and presence of Q waves were the frequently detected ECG abnormalities in a maximum number of elderly patients (46.53%). A similar finding was observed in elderly HF patients with reduced ejection fraction (p<0.001). The next common abnormality in the elderly with HFrEF was left axis deviation (LAD) (31.25%). However, LVH was seen in elderly patients with preserved ejection fraction (33.33%). Findings reported by Opadijo and Omotosho [5] mention LVH as the commonest ECG abnormality (68%) in patients with HF and HFrEF, followed by LAD.

Studies from Western countries have also shown that coronary artery disease, either alone or in combination with hypertension, appears to be the most common causative factor of heart failure. This may be because long-term chronic hypertension increases the risk of developing several conditions like LVH, myocardial infarction, heart failure, arrhythmia, and left atrial abnormalities. In the absence of coronary atherosclerotic disease, age-related decline in coronary reserve may also cause myocardial ischemia, hence contributing to additional diastolic abnormalities [6].

Another study by Karaye and Sani [7] (2008) showed LVH as the most common ECG abnormality found in 76 subjects (67.3%), followed by LAE in 58 patients (51.3%). Left ventricular hypertrophy was also the commonest abnormality among patients with preserved LVEF (50.0%) as well as in those with reduced LVEF (77.5%; p=0.0026).

In our study, atrial fibrillation (AF) was observed to be the most common form of arrhythmia present in 24.75% of HF patients and incidence was 25% among elderly patients with reduced ejection fraction. AF was reported in 23.82% of the patients with preserved ejection fraction. The difference in AF between the two groups was not significant (p=0.91). A study on electrocardiographic abnormalities in hospitalized elderly patients by Fariñas et al. [8] showed that AF was recorded in 16.9% of cases. Thomas et al. [4] reported no significant difference in the prevalence of AF in HF patients with preserved LVEF (19%) and reduced LVEF (10%) (p=0.09). The mean age of patients in this study was 56.5 years. 75% of the study sample were African-Americans and only 10% were Caucasians.

A study done by Opadijo and Omotosho [5] found that 7.3% of HF patients with reduced ejection fraction had AF. The mean age of the patients in their study was 57.3 years. However, a study conducted by Owan et al. [2] found the prevalence of AF was 41.3% in patients with heart failure with reduced ejection fraction and 28.5% in HF patients with normal LVEF (p<0.001). Bhatia et al. [3] also reported a higher prevalence of AF in both heart failure with reduced ejection fraction patients (31.8%) and patients of HF with preserved ejection fraction (23.6%) (p<0.001). The mean age of patients in Owan et al. [2] and Bhatia et al. [3] was above 70 years. All three studies [2-4] were carried out in the United States and Canada. 

In our study, QTc prolongation was seen in 19.8% of all elderly HF patients, 18.75% of the HFrEF group and 23.80% of the HFpEF group. The group difference was not statistically significant (p=0.60). 

A higher percentage of heart failure patients (51%) were reported to have a prolonged QTc by Vrtovec et al. [9]. Boccalandro et al. [10] also reported a prolonged mean QTc among HF patients (mean of 447 ± 33 ms) with an inverse relationship between the duration of QTc and the severity of HF. We had a lesser number of heart failure patients with prolonged QTc in our study. This was perhaps because the risk factors like female sex and LVH, which have been found to be independent predictors of prolonged QTc [11-13] were lesser represented in our study population, with females being (33%) and LVH present in 19% of patients only. 

Other isolated ECG abnormalities detected were tachyarrhythmias (9.9%), including sinus tachycardia, bradycardia and junctional bradycardia. Various blocks including right and left bundle branch blocks and first-degree heart blocks comprised 28.71%.

Limitations

This study used data from a single centre, which may have resulted in a very homogeneous population, hence the results obtained are not generalisable to other parts of India or the world. Holter monitoring was performed for only 24 hours after the admission. It is recommended that if no arrhythmia is detected within 24 hours and the patient manifests the symptoms, extending the duration to 48-72hours or repeat Holter monitoring is essential. Thus, the short duration of the monitoring in this study is considered a limitation. Extended Holter monitoring should be considered in persistently symptomatic patients, which was lacking in this study.

## Conclusions

Ischemic heart disease and hypertensive heart diseases are the most commonly reported etiological factors of heart failure in the elderly population of India, especially in the North. Patients with reduced ejection fraction had more ECG abnormalities. Atrial fibrillation was the commonest arrhythmia found in elderly HF patients. The difference in detecting the arrhythmias between the surface ECG and Holter monitoring was significant in patients with reduced EF (p=0.007) and is found to be higher in the latter. However, the difference was minor in elderly patients with preserved ejection fraction. Thus, Holter monitoring can be used as an additional modality for risk stratification of elderly heart failure patients with reduced ejection fraction.
